# Bone Metastasis as the Only Metastatic Site in a Patient with Pancreatic Cancer following Distal Pancreatectomy

**DOI:** 10.1155/2010/634975

**Published:** 2010-08-24

**Authors:** Muhammad Wasif Saif, Natalie Galanina, L. Ravage-Mass, Kristin Kaley, Daniel Cornfeld, Lynne Lamb, David Chhieng

**Affiliations:** ^1^Division of Hematology/Oncology, Department of Medicine, Columbia University College of Physicians and Surgeons, NY 10032, USA; ^2^GI Oncology Section, Division of Hematology/Oncology, Herbert Irving Comprehensive Cancer Center (HICCC), Columbia University, NY 10032, USA; ^3^Pancreas Center, New York Presbyterian Hospital, NY 10032, USA; ^4^School of Medicine, Yale University, New Haven, CT 06510, USA

## Abstract

Pancreatic cancer remains a challenge both diagnostically and therapeutically. The typical sites of metastases in pancreatic cancer include the liver and peritoneum. Other less common sites are the lung, brain, kidney, and bone. Skeletal metastases are less prevalent in occurrence but contribute to significant morbidity associated with pancreatic cancer. The prevalence of osseous metastases remains unknown but has been estimated to be between 5% and 20%. The most common osseous lesions are osteolytic in nature, but the osteoblastic ones are extremely rare. Here, we report an interesting case of pancreatic adenocarcinoma with exclusive bone metastases and discuss briefly the possible pathogenesis.

## 1. Introduction

The typical sites of metastases in pancreatic cancer include the liver and peritoneum. Other less common sites are the lung, brain, kidney, and bone [1, 2]. Skeletal metastases are less prevalent in occurrence but contribute to significant morbidity associated with pancreatic cancer. The prevalence of osseous metastases remains unknown but has been estimated to be between 5% and 20% [2]. The most common osseous lesions are osteolytic in nature, but the osteoblastic ones are extremely rare [3–6]. Here, we report an interesting case of pancreatic adenocarcinoma with exclusive bone metastases. 

**Figure 1:**
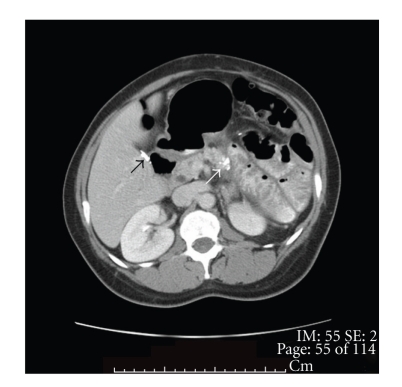
46-year-old woman with pancreatic cancer and prior distal pancreatectomy. CT scan with contrast through the pancreas. There is calcification at the cut surface of the pancreas (white arrow). No residual tumor is seen at this site. Cholecystectomy clips are present in the gall bladder fossa (black arrow).

**Figure 2:**
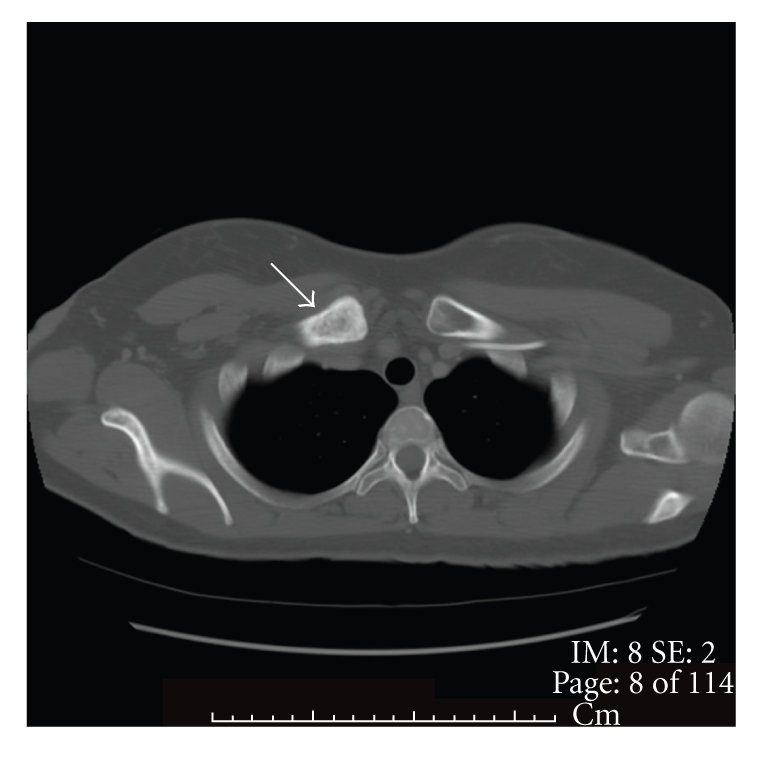
CT scan with contrast through the clavicle. There is increased sclerosis of the medial right clavicular head (white arrow) with a moth-eaten appearance of the cortex and medullary space. The differential diagnosis is metastasis or chronic osteomyelitis. Biopsy subsequently showed metastatic pancreatic cancer.

**Figure 3:**
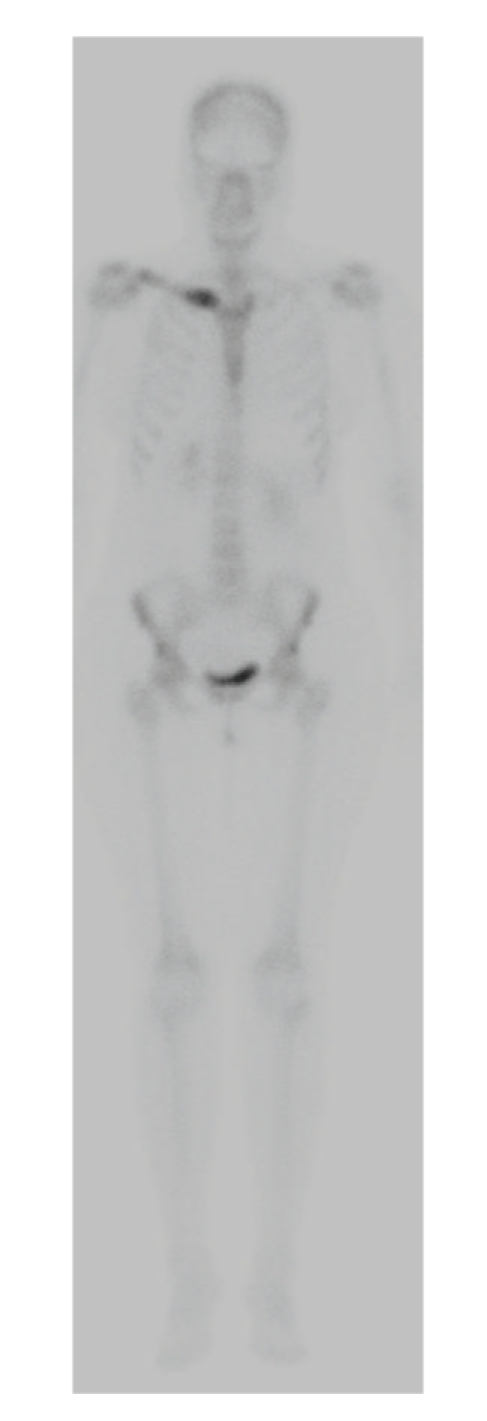
Frontal projection from a bone scan shows increased tracer uptake in the medial right clavicle, consistent with either metastatic disease or osteomyelitis. Biopsy confirmed metastatic pancreatic cancer.

**Figure 4:**
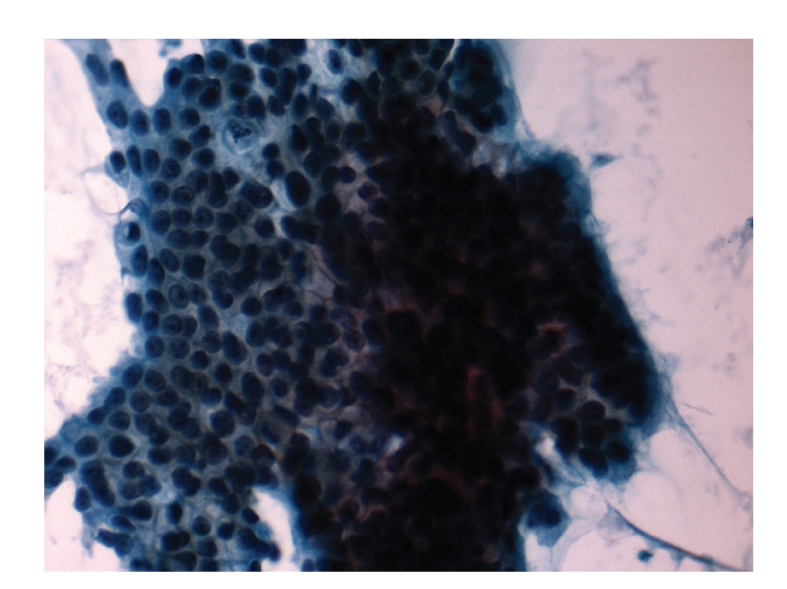
Histological findings of the clavicle biopsy showing metastases pancreatic cancer.

Our patient is a 46-year-old African American woman who was initially diagnosed with pancreatic adenocarcinoma in spring of 2007. A CT scan of chest/abdomen/pelvis revealed no evidence of metastatic disease, including bones (Figure 1). She underwent a distal pancreatectomy and splenectomy. The pathology showed pancreatic ductal adenocarcinoma and evidence of lymphovascular invasion with 3 of 18 lymph nodes involved. Her CA19-9 was within normal limits (<37 U/L), and CEA was slightly elevated at 5.6 U/L. Thus, the patient was staged as IIB. She was subsequently treated with six cycles of gemcitabine (Gemzar; Eli Lilly). Patient was in clinical remission until July of 2009 when she developed right clavicle pain. A repeat CT scan showed increased sclerosis of the medial right clavicular head (Figure 2) with a moth-eaten appearance of the cortex and medullary space. The differential diagnosis included metastasis or chronic osteomyelitis. This was followed by a PET scan that showed increased tracer uptake in the medial right clavicle, consistent with either metastatic disease or osteomyelitis (Figure 3). A fine needle biopsy of the right clavicle was performed which confirmed metastatic pancreatic cancer (Figure 4). Upon further investigation, patient did notice pain in the area of the bone disease on the right neck area. The patient was started on Zoledronic Acid (Zometa) and referred to a radiation oncologist. She received a palliative course of radiation therapy to the affected clavicle for 2 weeks. As she was near completing her course of radiation therapy to the clavicle, she complained of new left hip pain. An MR imaging revealed radiographic evidence of focal disease in the left ischium, acetabulum, and femur. She was given an additional dose of ten fractions of radiation therapy to the hip area. Restaging CT showed interval progression of metastatic bony disease manifested by a new lesion in the left sacrum. Patient was started on modified FOLFOX6 consisting of 2 weekly cycles of oxaliplatin 85 mg m^2^ i.v. over 2 h, together with leucovorin 400 mg m^2^ over 2 h, 5-fluorouracil (5-FU) 400 mg m^2^, and bolus, followed by a 46-h infusion of 5-FU at 2.4 g m^2^. After a 12-week therapy with FOFOX regimen, the patient had a repeat whole body bone scan which showed multiple new foci of increased uptake as follows: at the base of the skull involving the cervical spine, L2 and L3 vertebrae, approximate left 5th and 8th ribs/costovertebral regions of the thoracic spine, humeri (R > *L*), proximal right radius/ulna, upper sacrum, multiple areas in the left bony pelvis, femora bilaterally, and left knee; previously noted increased uptake along the right clavicle appeared more intense compared to the baseline study. The relentless progression of the disease was also reflected by a significant increase in Carcinoembryonic Antigen (CEA) rising from 136 ng/ml (10/2009) to 172 ng/ml (12/2009) (normal range <3 ng/ml); CA19.9 remained <0.8 U/ml. Interestingly, there had been no evidence of soft tissue disease either by the CT (chest/abdomen/pelvis) or brain MRI. The FOLFOX therapy was discontinued; her CEA continued to rise reaching 338 ng/ml in 02/2010. Currently, the patient is on best supportive care.

Most patients who develop osseous metastasis have already concomitant visceral metastases. Our patient developed metastatic osteoblastic lesion of the clavicle following Whipple procedure, and the diagnosis was confirmed by biopsy of the bone. The uniqueness of this case lies in the osteoblastic nature and in the absence of visceral metastasis. In a retrospective chart review, patients with bone metastases uniformly exhibited at least one or more site of disease with the most common one being the liver followed by the peritoneum and lungs [1, 6, 7]. One case has reported lesion in L3 vertebra [2], but we believe that our patient is the first report of pancreatic cancer confined to a distant bone metastasis (clavicle) as the only site without overt visceral metastases. 

Bone involvement in patients with pancreatic cancer may result from direct posterior extension of the primary tumor with destruction of the bodies of one or more upper lumbar vertebrae in the former case [2, 8] while haematogenous spread may be responsible for our report [3]. Review of literature also suggests various cytokines such as interleukin-6 [9], vascular endothelial growth factor (VEGF), and parathyroid hormone-related protein (PTHrP) which exert a promotive effect on bone resorption and may play a pivotal role in the development of intraosseous progression of pancreatic adenocarcinoma [10]. Both osteolytic and osteoblastic lesions have been described, suggesting multiple mediators of bone metastases. Transforming growth factor beta (TGF-b), IL-11, and matrix metalloproteinases have been shown to stimulate osteoblastic activity in other tumor types [10]. Additional factors such as ethnic, genetic, and biologic variables that determine the “homing” and proliferation of tumor cells remain to be elucidated. 

Pancreatic and gastric malignancies demonstrate CEA level elevations in just over 50% of cases [11]. In a study conducted by Kokhanenko et al. [12], CA19-9 and CEA were studied in 685 examinations for PC, 68 chronic pseudotumorous pancreatitis and 24 intestinal cancer at other sites since 1995. Tumor resection for PC was carried out in 31, conservative treatment in 67, chemotherapy in 56, and radiotherapy in 29 cases. In CA19-9 examinations, diagnostic sensitivity was 90.2 specificity was 72.1 effectiveness was 85.3% while in CEA determinations, 82.5, 30.9, and 68.5%, respectively. CA19-9 and CEA levels proved to be prognostic factors of survival. An inverse correlation was observed between median survival and tumor marker concentrations: higher basal (preoperative) level of marker in blood was matched by lower median survival. A similar relationship was identified for CEA: 5–10—14.2 months, 10.1–20 ng/ml—8.0 months, 20.1–30 ng/ml—3.9 months, and more than 30 ng/ml—4.8 months. In our practice, we measure both markers at baseline and follow the one which was found elevated at the time of initial assessment.

In conclusion, this case emphasizes individual variability in the incidence of bone metastases secondary to adenocarcinoma of the exocrine pancreas in order to help clinicians recognize and treat bone metastases early to improve quality of life and reduce morbidity associated with the progression of the disease.
